# Coronary artery bypass grafting hemodynamics and anastomosis design: a biomedical engineering review

**DOI:** 10.1186/1475-925X-12-129

**Published:** 2013-12-13

**Authors:** Dhanjoo N Ghista, Foad Kabinejadian

**Affiliations:** 1 Southern Ozarks Alliance for Rural Development, Willow Springs, MO 65793, USA; 2 Department of Biomedical Engineering, National University of Singapore, 9 Engineering Drive 1, Block EA #03-12, Singapore 117576, Singapore

**Keywords:** Coronary artery bypass graft (CABG), Anastomosis, Stenosis, Hemodynamic, Wall shear stress, Intimal hyperplasia, End-to-side, Side-to-side

## Abstract

In this paper, coronary arterial bypass grafting hemodynamics and anastomosis designs are reviewed. The paper specifically addresses the biomechanical factors for enhancement of the patency of coronary artery bypass grafts (CABGs). Stenosis of distal anastomosis, caused by thrombosis and intimal hyperplasia (IH), is the major cause of failure of CABGs. Strong correlations have been established between the hemodynamics and vessel wall biomechanical factors and the initiation and development of IH and thrombus formation. Accordingly, several investigations have been conducted and numerous anastomotic geometries and devices have been designed to better regulate the blood flow fields and distribution of hemodynamic parameters and biomechanical factors at the distal anastomosis, in order to enhance the patency of CABGs. Enhancement of longevity and patency rate of CABGs can eliminate the need for re-operation and can significantly lower morbidity, and thereby reduces medical costs for patients suffering from coronary stenosis. This invited review focuses on various endeavors made thus far to design a patency-enhancing optimized anastomotic configuration for the distal junction of CABGs.

## Introduction

Coronary artery disease (CAD) is the leading cause of death globally, and is expected to account for 14.2% of all deaths by 2030 [1]. According to the statistics from the American Heart Association, mortality data show that cardiovascular disease, as the underlying cause of death, accounted for 34.3% of all (1 of every 2.9) deaths; in particular, coronary heart disease caused approximately 1 of every 6 deaths in 2006 in the United States [2].

Several alternative treatments exist for CAD, including medical therapy, rotablation, endarterectomy, percutaneous coronary intervention (PCI) or balloon angioplasty, stenting, and coronary arterial bypass grafting (CABG). Depending on the severity, number and position of atherosclerotic lesions and the clinical history of the patient, any of the above mentioned treatments may be chosen. For high-risk patients, such as those with left main coronary artery (LMCA) disease, severe three-coronary vessel disease, severe ventricular dysfunction (i.e., low ejection fraction), and diabetes mellitus, CABG is the preferred treatment [3]. In general, the greater the extent of coronary atherosclerosis and its diffuseness, the more compelling the choice of CABG, particularly if the left ventricle function is depressed [4].

**Figure 1:**
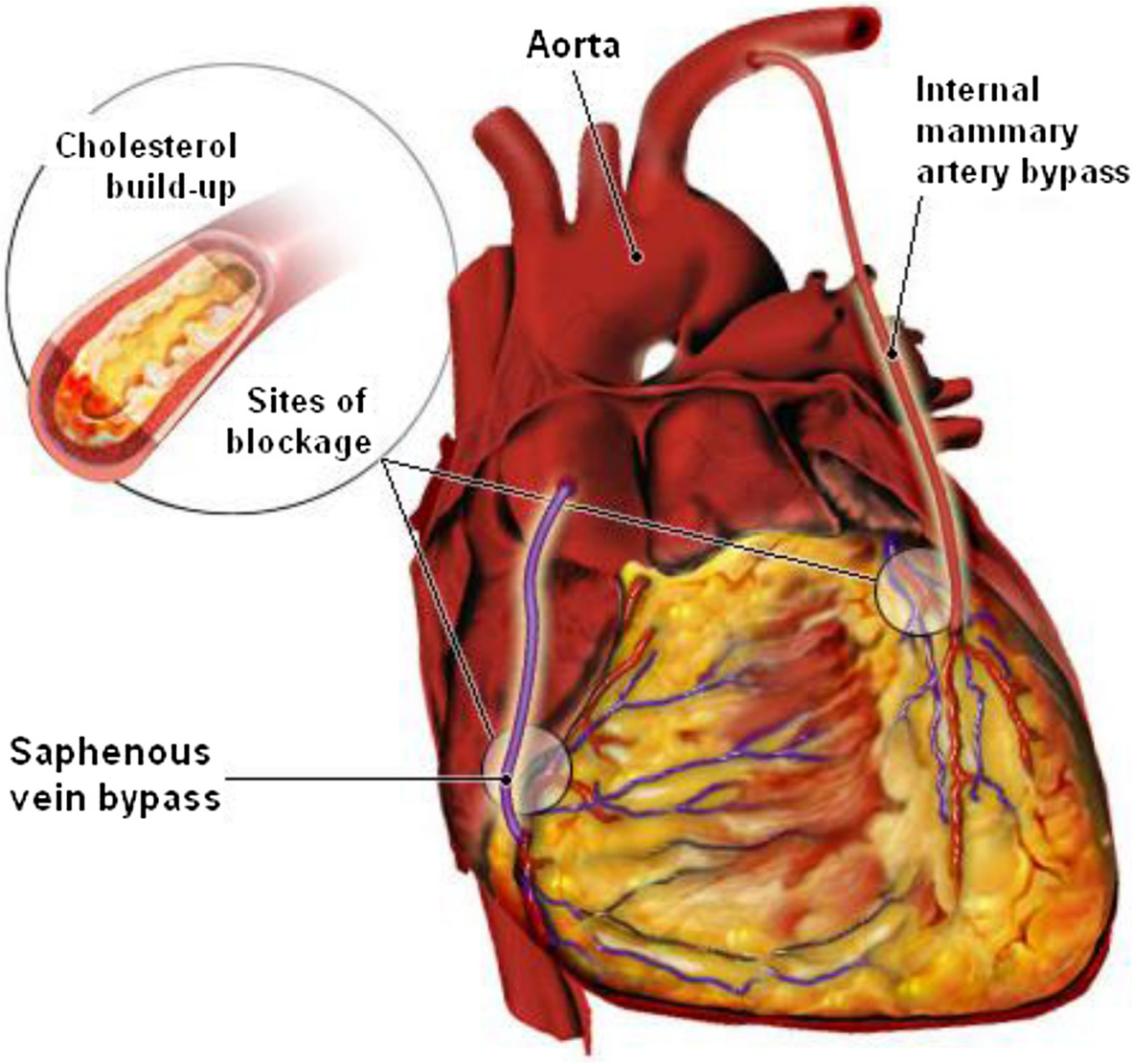
**Illustration of coronary arterial bypass grafting.** A saphenous vein graft is anastomosed proximally to aorta and distally to downstream of the stenosis of the right coronary artery (RCA). The internal mammary artery (IMA) which branches from aorta is anastomosed to the left anterior descending (LAD) coronary artery.

CABG is a surgical procedure performed to graft arteries or veins from the patient’s body or synthetic conduits to the occluded coronary arteries in order to bypass the atherosclerotic narrowing and improve the blood supply to the coronary circulation, for nourishing the myocardium. Figure 1 illustrates both arterial and venous grafts, each bypassing a coronary blockage (stenosis) formed by cholesterol build-ups.

Although CABG is extremely effective for symptomatic relief and prognostic improvement in CAD and is the preferred remedy for high-risk patients, it is not devoid of complications and the long-term benefits are directly related to continuing conduit patency. Approximately 10–15% of vein grafts occlude during the first year after operation [5]. About half of the vein grafts are only effective for a period of 5 to 10 years [6,7]. By 10 years after surgery, about 60% of vein grafts are patent, only 50% of which remain free of significant stenosis [8].

Early graft failure (within 30 days) of bypass grafts is attributable to surgical technical errors and resulting thrombosis, while late graft failures are mainly caused by progression of atherosclerosis and intimal hyperplasia (IH) [9]. Various studies have found IH to be the major cause of graft stenosis [10]. IH is the abnormal, continued proliferation and overgrowth of smooth muscle cells (SMCs) in response to endothelial injury or dysfunction. Although the exact mechanism and pathophysiology of IH remains an enigma, there are indications that both biological and biomechanical factors are involved, which include endothelial injury [11], platelet activation [12], disturbed local hemodynamics [13,14], compliance mismatch between the graft and host vessel [15], and interactions between blood and graft material [16].

Among the abovementioned factors, hemodynamic parameters (HPs) are believed to be highly important [17,18] in the genesis and development of IH. It has been shown that in end-to-side (ETS) graft–artery configurations, IH develops predominantly at the toe and heel of the anastomosis and on the artery bed across the junction where disturbed flow patterns and hemodynamic factors are observed [17,19]. On the basis of this focal distribution of intimal thickening (IT), disturbed flow patterns and the associated hemodynamic factors have been correlated with the onset and progression of atherosclerosis and distal anastomotic intimal hyperplasia (DAIH) [19,20]. Among the important hemodynamic factors are wall shear stress (WSS), spatial and temporal gradients of WSS, and oscillatory shear index (OSI).

Accordingly, several investigations have been conducted and different anastomotic geometries and devices have been designed to improve the flow fields and HPs distribution at ETS anastomosis, in order to enhance the graft patency. These investigations include studies on the effects of geometrical factors, such as anastomotic angle [21-27], modified configuration of distal anastomosis [28-31], graft-to-host artery diameter ratio [32-34], and out-of-plane graft [35-37], and effects of stenosis severity and proximal artery flow [38,39], irregularities of venous graft wall (due to venous valve sinus) [40], and distance of grafting (i.e., the distance of anastomosis from the occluded site) [41]. Considerable efforts towards attaining an optimal patency-enhancing CABG anastomotic configuration have been made, and continue to be made by investigators. This is because enhancement of the longevity and patency rate of CABGs (by means of an optimal anastomotic configuration) can result in considerable improvement in the left ventricular contractility index and ejection fraction of patients with CAD [42], elimination of the need for re-operation, reduced medical costs for patients suffering from coronary stenosis, and significantly lower morbidity.

Accordingly, this paper reviews the theories on bypass graft failure and its causative biological and biomechanical factors, followed by various attempts to design an optimal anastomotic configuration for the distal CABG anastomosis. This review illuminates the impact of CABG layout designs towards obtaining higher long-term graft patency rates and the benefit of superior anastomotic designs for the improvement of global ejection fraction of patients with coronary artery disease.

The first section of this review covers the studies correlating different biological and biomechanical factors to initiation and progression of atherosclerosis and IH. The majority of the investigations on biological factors are in vivo studies along with complementing in vitro investigations. On the other hand, computational simulations of blood flow along with numerous in vitro and in vivo investigations constitute the studies of the biomechanical factors and hemodynamic parameters, and have provided strong evidence on the influence of these factors on initiation and onset of IH.

The second section of this review elaborates on the various attempts to design an optimal anastomotic configuration for CABG. These attempts include adjustments of anastomotic angle and graft-to-host artery ratio, design of different cuffed and patched anastomotic configurations, and design and development of other novel configurations (such as the coupled sequential anastomoses) and synthetic devices.

Finally, the current state of the art of CABG anastomotic configurations is discussed and future directions are suggested.

## Theories on bypass graft failure

According to angiography and histological examinations, common graft failure modes include acute thrombosis, IH, and onset of progressive atherosclerosis [8,43,44]. Thrombosis is the formation of a blood clot (thrombus) in arteries or veins, as a result of low blood velocity within a graft caused by a flow-limiting stenosis or high-shear rates caused by a jet-type flow [45]. Atherosclerosis is a disease of the arteries in which fatty material and plaque are deposited in the wall of an artery, resulting in narrowing of the arterial lumen and eventual impairment of blood flow. The predominant theory suggests that atherosclerosis develops in response to injury [46]; however, there is no consensus as to whether this injury is mechanical, chemical, immunological, or a combination of these and other factors.

IH, the major cause of graft failure, is the abnormal increase in the number of endothelial cells (ECs) and thickening of the tunica intima of a blood vessel. IH was first described by Carrel and Guthrie [47], who observed that within a few days after arterial vein graft implantation, the anastomotic stitches happened to be covered with a material similar to the normal endothelium.

Although the pathological mechanisms responsible for the development of IH and atherosclerosis have not been fully elucidated, many theories have been hypothesized, indicating the involvement of both biological and biomechanical factors, some of which are briefly discussed in the following sections.

### Biological factors

The endothelial cells sense the mechanical forces acting on them through mechanotransducers [48]. The presence of abnormal mechanical forces on the endothelium leads to some biological effects, which can trigger excessive release of mitogens and growth factors and lead to subsequent SMC proliferation and excessive platelet aggregation [49]. Monocytes and cholesterol accumulate in the intima, creating foam cells. Monocytes become macrophages within the intima, resulting in formation of an atherosclerotic plaque [50].

Older plaques can develop into a calcified lesion or nodule. The origin or mechanism of the calcification is not precisely known, but it appears to be associated with healed plaque. The artery with a rigid matrix is unable to remodel, causing further cellular proliferation to push the fibrous cap out into the lumen. The rupture of this fibrous cap exposes tissue factors and collagen to the blood, forming a nidus for thrombus formation and consequent restenosis.

For more details, see the reviews of hypothesized theories on molecular mechanisms and biological factors involved in the development of IH [6,48,51-56].

### Vascular injury

During the surgery, endothelium and SMCs of the recipient artery and the autogenous graft are injured in several ways: grasping of the instruments used to harvest the graft; routinely applied high intra-luminal pressure to check for the graft leakage; and sutures for the construction of the anastomoses. In addition, due to implantation of the vein graft in the arterial circulation, the grafted vein is exposed to high pressures and high blood flow causing further damage to the venous endothelium [57].

**Figure 2:**
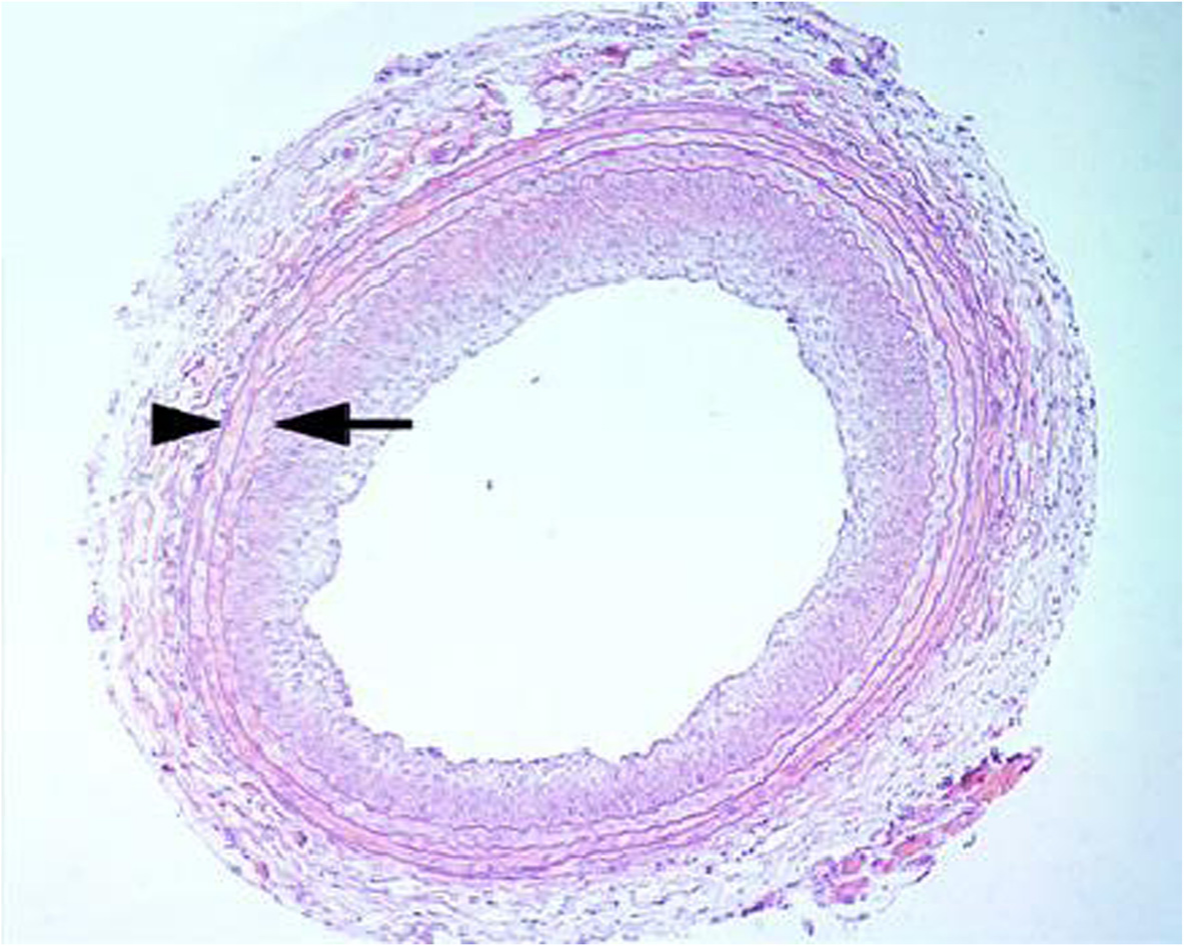
**Injury-induced intimal hyperplasia.** A tissue section of a rat’s common carotid artery: Intimal hyperplasia 14 days after treatment with angioplasty balloon. The locations of the internal and external elastic lamina are indicated by an arrow and an arrowhead, respectively (taken from [60] with permission).

The injured endothelium is less capable of producing anti-proliferative products [6], and the physiological balance in the vessel wall is further disturbed by the release of intracellular growth factors from injured medial SMCs [6,58]. It has been demonstrated that acute injury to the intima and media can produce hyperplasia and SMCs proliferation, which occur at a rate proportional to the degree of the injury [11,49]. It is also observed that IH forms around the injury sites after balloon angioplasty [59] (Figure 2). These suggest the involvement of a wound healing process in IH formation.

For further details on pathophysiological mechanisms following vascular injury, see references [61-63].

### Compliance mismatch

**Figure 3:**
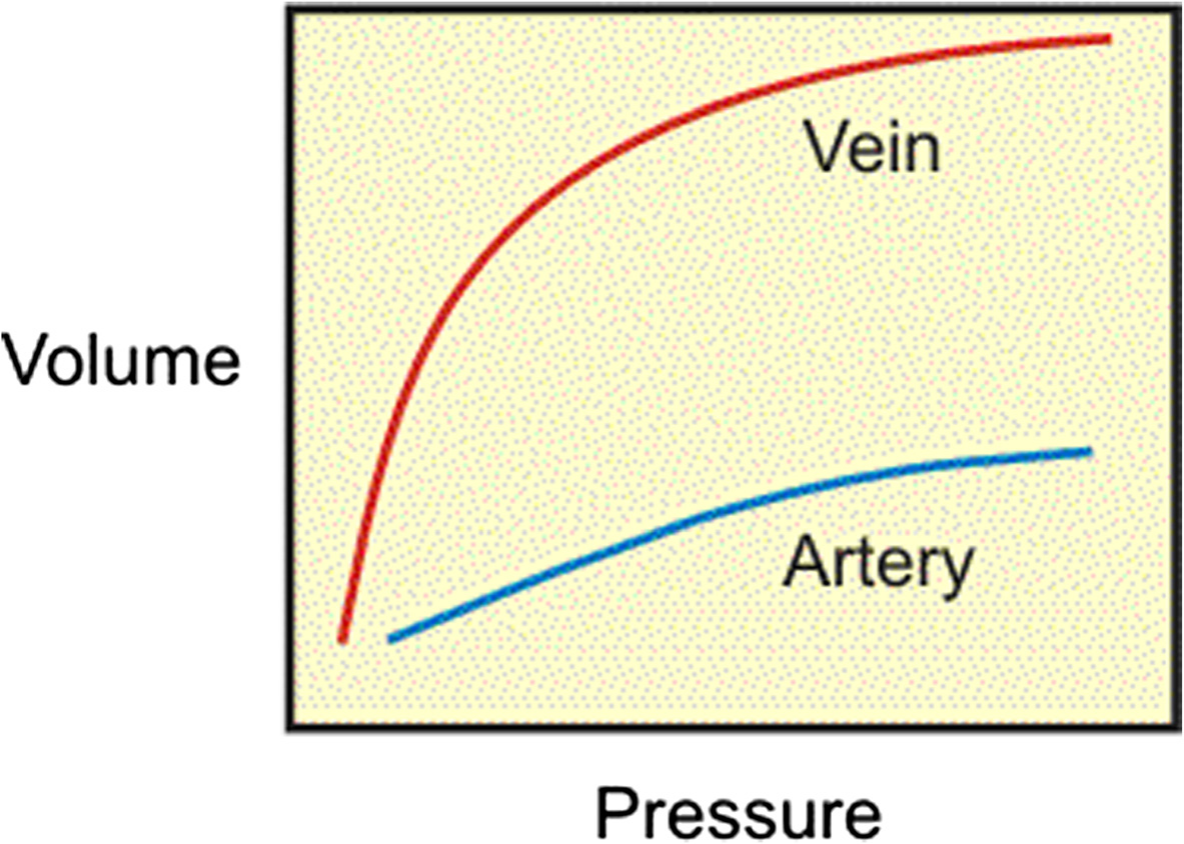
**Volume-Pressure curves for vein and artery.** Schematic volume-pressure curves for artery and vein (the slope of the curve indicates the compliance). Compliance of a vein is greater than arterial compliance at low pressures and smaller than that at high pressures.

The ability of a blood vessel to expand and contract passively with changes in pressure is an important function of large arteries and veins. Compliance is a measure of the tendency of a hollow organ to resist recoil toward its original dimensions upon removal of a distending or compressing force. In other words, the ability of a vessel to distend and increase volume with increasing transmural (inside minus outside) pressure is the vessel compliance (C), which is defined as:

(1) $$C=\frac{\mathrm{\Delta V}}{\mathrm{\Delta P}}$$

where ∆*V* is change in volume and ∆*P* is change in transmural pressure. It is usually assumed that an increase in the volume of a blood vessel segment is almost exclusively caused by an increase in its radius since elongation is negligible in-vivo [64]. Thus, compliance can be estimated through the variation in cross-sectional area, and the above relation can be rewritten as [65]:

(2) $$\mathrm{Cross}-\mathrm{sectional}\;\mathrm{compliance}\;C_{C}=\frac{\pi \left({D_{s}}^{2}-{D_{d}}^{2}\right)}{4P_{P}}$$

where *D*_*d*_ is diastolic diameter, *D*_*s*_ is systolic diameter, and *P*_*P*_ is the pulse pressure. Veins have a higher compliance than arteries due to their thinner walls. A schematic volume-pressure relationship for an artery and vein is depicted in Figure 3. Two important characteristics stand out. Firstly, the curves are not linear (slopes are not constant); this is because the blood vessel wall is a heterogeneous tissue. Therefore, compliance decreases at higher pressure and volume (i.e., vessels become stiffer at higher pressures and volumes). Secondly, although at low pressure the compliance of a vein is about 10 to 20 times greater than that of an artery, at high pressure and volume, venous compliance (slope of volume-pressure curve) becomes smaller than arterial compliance. In case of arterial bypass, a mismatch in compliance between the vascular graft and the host artery is regarded as a mechanical factor which is detrimental to graft patency.

**Figure 4:**
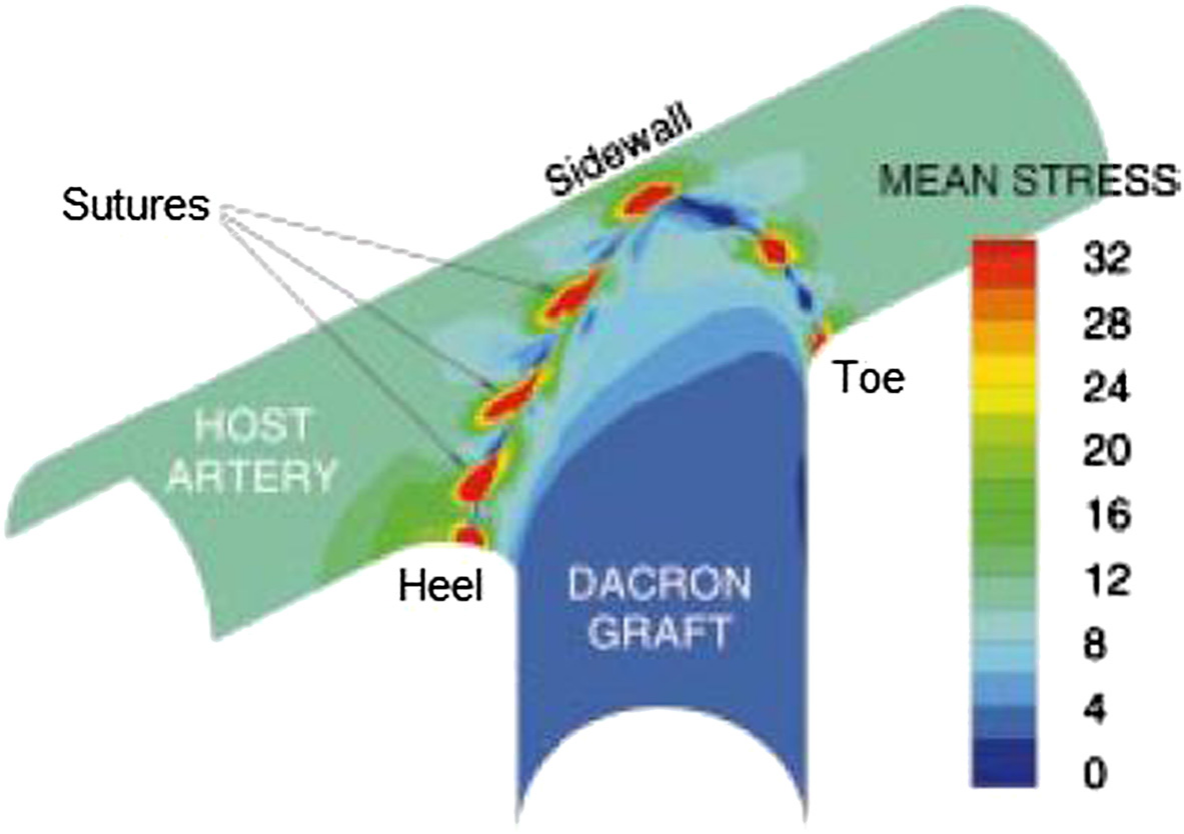
**Intramural stress distribution at a conventional ETS anastomosis.** Contour of mean intramural stress (normalized by the inflating pressure) at the anastomosis of a conventional ETS graft-artery junction. The suture-induced stress concentrations range from 3 to 36 times the stress values along the distal host artery (taken from [15] with permission).

It has been shown in vivo that a higher degree of compliance mismatch between the graft and the artery results in a greater amount of suture-line IH formation in ETS anastomoses [17,66,67], but not in end-to-end (ETE) junctions [68]. Following these observations, Ballyk et al. [15] performed a large-strain finite-element analysis of vascular wall mechanics to compare the influence of compliance mismatch on intramural stresses in ETE versus ETS anastomoses. They found out that increased compliance mismatch increases stresses in ETS junctions (Figure 4), but, it has little influence on stresses in ETE junctions, suggesting that the proliferative influence of increased compliance mismatch on suture-line hyperplasia in ETS anastomoses can be explained by the resulted increase in intramural stresses. As high intramural stresses have generally been observed at suture-lines in both ETS and ETE junctions, it has been suggested that elevated suture-line intramural stresses might be an important proliferative stimulus for IH formation in vascular reconstructions [15], and that IH might be a response to the mechanical injury caused by stretch and high intramural stresses as a result of compliance mismatch [67].

Although the detailed mechanism for stress-induced IT is not completely understood, it has been shown that endothelial cells respond to stretch stimuli by producing transcription factors which stimulate their replication [69] and chemotactic factors and mitogens which cause smooth muscle cells to migrate and proliferate [70].

**Figure 5:**
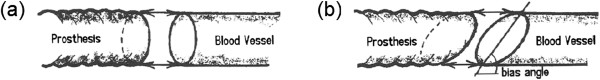
**Geometric compliance matching.** The graft constructed with an elliptic cross-section **(b)** develops peak stresses that are orders of magnitude lower than those developed with conventional ETE configurations **(a)**. Circular grafts and vessels cut on a bias (bevelled end) provide an increased anastomotic junction perimeter which can reduce stress concentrations by distributing the loading force among a potentially greater number of sutures. The introduction of matching geometric compliance that dominates at the anastomotic junction minimizes the consequences of material mismatch between graft and vessel and has the potential to reduce the suture line stress (taken from [71] with permission).

Compliance mismatch is not only between the stiffer bypass graft and the artery, but also at the anastomosis itself between the suture and the graft on one side and between the suture and the artery on the other side, forming a para-anastomotic hyper-compliant zone and elevating intramural stresses. Different surgical techniques have been suggested to reduce the level of intramural stresses (elevated by compliance mismatch) and the amount of consequent IH (e.g., by geometric compliance matching (Figure 5) [71] or by using an external stent/sheath [72,73]). However, it seems that compliance mismatch alone, without trauma caused by suturing, causes only limited amounts of IH formation [74]. Thus, in order to alleviate the trauma in anastomosis, alternatives to sutures have been suggested, including biological glues, clips, clamps, magnetic vascular positioners, and laser generated solders, some of which have shown promising results [75-78]; yet, further work is required before they can become applicable for routine use.

Although compliance mismatch has been reported to cause trapping of micro-particles near the wall downstream the anastomosis [79] and to disturb the protein transport process [80] in ETE junctions, computational studies of ETS anastomosis models have illustrated that compliance mismatch has only minor effects on local hemodynamic factors [18,81].

### Hemodynamic factors

**Figure 6:**
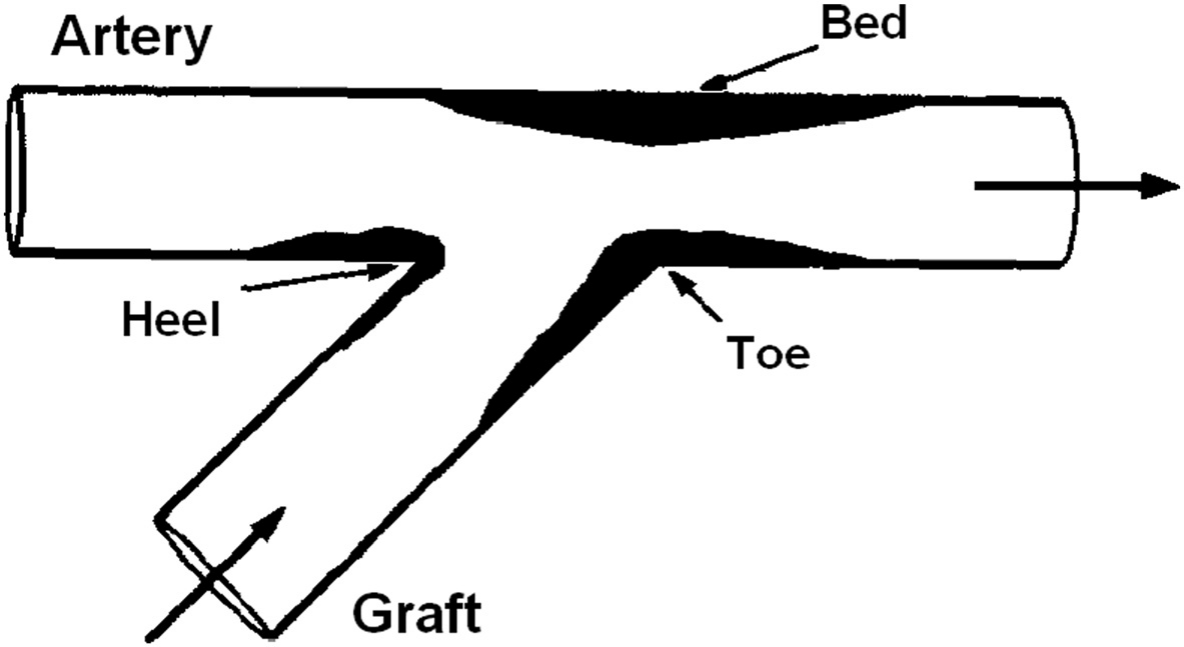
**Spatial distribution of intimal hyperplasia in an end-to-side distal anastomosis.** Outline of the spatial distribution of IH in an ETS distal anastomosis: IH occurs preferentially around the suture-line (especially at the toe and heel of the anastomosis) and on the bed of the host artery (taken from [84] with permission).

In vivo observations indicate that in an ETS graft-artery anastomotic configuration, IH occurs preferentially around the suture-line (especially at the toe and heel of the anastomosis), and also develops on the bed of the host artery across the junction [17,19,26,67,82,83] (Figure 6).

**Figure 7:**
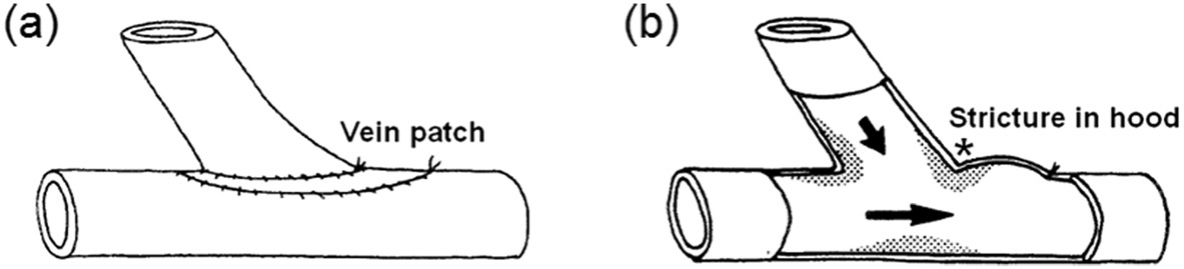
**In vivo evidence that geometry of ETS anastomosis causes IH. (a)** ETS distal anastomosis with a vein patch **(b)** a section of distal ETS anastomosis; creation of an arbitrary stricture at the hood to simulate the toe (*) results in transferring the IH from the suture-line at the anastomotic toe to the stricture at the hood (physiologic toe); arrows indicate direction of blood flow (taken from [83]).

Arterial floor IT is attributable to altered flow conditions [17]. Although it has been suggested that suture-line IT might be related to vascular healing, an in vivo study by Sottuirai [83] has shown opposing results. He investigated the role of anastomotic configuration, using autogenous femoro-femoral bypass with ETS configuration and ETE interposition graft in canine models. Since compliance mismatch is not an issue in the autogenous femoro-femoral bypass, for DAIH which exclusively occurred in the ETS (and not in the ETE) distal anastomoses, the geometry of the distal anastomosis has been concluded to be the logical causal factor. Moreover, creation of an arbitrary stricture on an extended hood of the arterial graft, to function as the “physiologic toe”, resulted in transferring DAIH to the site of the graft stricture (Figure 7). This attests to the fact that it is the ETS anastomotic configuration and its unnatural flow conditions, and not the trauma along the suture-line, which contributes to DAIH formation (more details are given in the next section on “Disturbed flow patterns associated with distal ETS anastomosis”).

Several investigations have been conducted to better understand the relationship between blood flow-based stresses acting on the walls and IT in bypass grafts [13,17,19,85-88]. Subsequently, multiple hemodynamic parameters have been associated with occlusive formations in arterial bypass grafts and other branching blood vessel configurations, as follows.

#### Safe bandwidth of WSS

Endothelial cells are constantly exposed to shear stress, induced by blood flow. Endothelial shear stress (i.e., WSS or ***τ***_*w*_) is the product of dynamic viscosity (*μ*) and shear rate ($\dot{\gamma }=\partial U/\partial r$) of blood at the vessel wall:

(3) $$\tau_{w}={\left(-\mu \frac{\partial U_{\mathrm{z\theta}}}{\partial r}\right|}_{r=R}$$

where ***U***_*zɵ*_ is the velocity component parallel to the vessel wall, *r* is the radial axis, and *R* is the radius of the blood vessel.

The existence of a safe bandwidth of WSS has been suggested by Kleinstreuer et al. [89] to explain the localization of atherosclerotic plaques and IH, based on two contradictory hypotheses: (i) high shear stress theory and (ii) low shear stress theory.

By experimental exposure of endothelium to high shear stresses, Fry [90,91] showed that a sufficiently high shear stress level would induce endothelial injury and promote the development of lesions, which were postulated to increase the permeability of endothelium and to alter the transport of molecular species across the endothelial barrier into the arterial wall, resulting in plaque formation.

On the other hand, Caro et al. [92], observed that atherosclerotic lesions occur along the inner wall of arterial curvature, where low shear stress exists. Hence, they proposed that, due to low WSS and enhanced particle residence time in flow separation and flow recirculation zones, excess cholesterol is deposited on the surface of the lumen, initiating atheroma growth, while in the regions of moderately high WSS, more cholesterol is washed away by the blood flow.

Hence, combining the abovementioned opposing theories, it is suggested that there exists a safe bandwidth of WSS and the wall shear that falls outside of this range will result in plaque formation. This hypothesis has successfully determined the sites and growth patterns of atherosclerotic lesions and IH in several arterial bifurcations and bypass graft configurations, respectively [93,94]. Moreover, study of numerical results of simulation of blood flow in the human aortic arch has suggested preferential development of early atherosclerotic lesions in regions of extreme (either maxima or minima) WSS and pressure [95]. In addition, it has been reported that the ECs in both low and high shear regions experience structural and functional abnormalities [96], thereby supporting the hypothesis of “safe bandwidth of WSS”.

#### Low-magnitude high-oscillatory WSS

Based on the theory of Caro et al. [92], it is the “shear-dependent mass transfer” which is responsible for atheroma development and IT. Low shear stress acting on the vessel wall has been introduced as the key hemodynamic factor involved in the localization of IT, due to significant correlations found between the preferred sites of IT and the regions of slow recirculation flow (i.e., long particle residence time) with low WSS [14,88,97,98].

Morinaga et al. [99] investigated IT occurrence in autogenous vein grafts in dogs, by comparing the conditions of high flow rate and low WSS with low flow rate and high WSS. A comparatively significant intimal thickness was observed in high flow rate and low WSS condition, revealing that WSS, and not the rate of flow, is the essential hemodynamic factor related to IH.

Ku et al. [100] found a positive correlation between plaque location and low, oscillating shear stress, indicating that marked oscillations in the direction of wall shear may enhance atherogenesis. Consequently, they put forward the concept of “oscillatory shear index” to quantify the oscillatory nature of WSS. Based on its modified definition (Equation 4) [101], the OSI value varies between 0 and 0.5, where 0 corresponds to the regions experiencing no reverse flow, and 0.5 is for the case of fully oscillatory flow without net forward flow.

(4) $$\mathrm{OSI}=\frac{1}{2}\left(1-\frac{\left|\int_{0}^{T}\tau_{w}^{\to }\mathrm{dt}\right|}{\int_{0}^{T}\left|\tau_{w}^{\to }\right|\mathrm{dt}}\right)$$

where *T* is the time period of a cardiac cycle and $\tau_{w}^{\to }$ is the WSS vector.

Li and Rittgers [102] compared the mechanical factors, obtained from in vitro study of pulsatile flow in a model of the distal ETS anastomosis of an arterial bypass graft, with histological findings of IH formation from earlier canine studies. Theeir results suggest that regions exposed to a combination of low-mean WSS and high-OSI may be most prone to IH formation. The same conclusion was obtained using in vitro preconditioned human umbilical vein ECs [103]. Besides, similar correlations of the hemodynamic parameters and sites of IT formation were observed by Zhang et al. [104] in a computational investigation of blood flow in a complete coronary artery bypass model.

Both low-mean shear and oscillatory shear stress contribute to an increased near-wall particle residence time, which may alter the mass transport of atherogenic substances to the vessel wall and increase the probability of deposition of platelets and macrophages, resulting in IT.

#### High gradients of WSS

**Figure 8:**
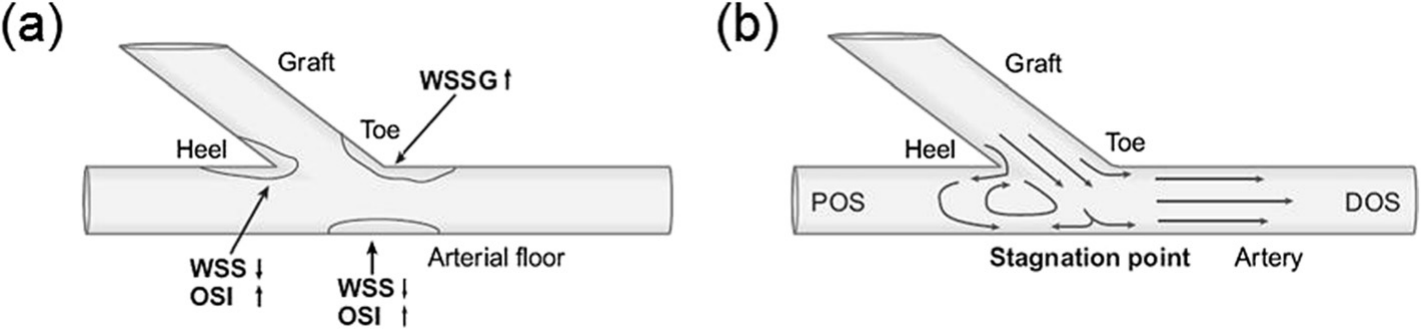
**Typical flow patterns and HPs distribution in a distal ETS anastomosis. (a)** Outline of the typical spatial distribution of HPs and IT, and **(b)** flow patterns in the distal ETS anastomosis of arterial bypass grafts. A stagnation point forms on the arterial bed due to the bifurcation of the graft flow into the proximal and distal outlet segments (POS and DOS) of the coronary artery after impinging on the arterial bed (adopted from [106] with permission).

In an ETS graft-artery anastomosis, the floor typically experiences low oscillating WSS, due to the presence of a moving stagnation point during the cardiac cycle, as shown in Figure 8. However, this low-WSS–high-OSI hypothesis does not explain IH formation at the toe of the anastomosis (as the WSS is neither low nor oscillating at this location). Consequently, it has been postulated that the large spatial WSS gradient (WSSG), which is mainly observed at the toe of the anastomosis, induces morphological and functional changes in the endothelium that contribute to elevated wall permeability and hence possible atherosclerotic lesions [105]. The spatial gradient of WSS represents the non-uniformity of the force distribution on the endothelium and implies a stretching force applied on the ECs, which can create local deformation of ECs and increase the wall permeability, leading to IH [28,84].

Moreover, the local WSSG is suggested as the single best indicator of non-uniform flow fields leading to atherogenesis [107]. Based on the biological evidence that non-uniform hemodynamic factors trigger an increase in wall permeability, Lei et al. [108] introduced an equation for wall permeability as a function of local WSSG magnitude; by employing the aorto-celiac junction of rabbits as a representative atherosclerotic model, their experimentally validated computer simulation model for enhanced LDL transport into the arterial wall showed that the WSSG is a reliable predictor of critical atherogenic sites in branching arteries [108]. Besides, it has been observed that IH tends to develop at sites having high spatial and temporal gradients in WSS [84].

#### Disturbed flow patterns associated with distal ETS anastomosis

The configuration of distal ETS anastomosis is not naturally present in the arterial system (except for the patent ductus arteriosus). Although an ETS anastomosis is basically a bifurcation, it is different from naturally occurring blood vessel bifurcations. The angle between the daughter vessels is effectively obtuse and the flow division between the daughter branches in anastomoses can vary widely which has a significant impact on the hemodynamics [45]. A distal ETS anastomosis is characterized by abnormal flow conditions, including flow oscillation at the heel, impact on the artery floor, and flow separation at the toe.

Typically, there is a low-WSS region at the heel, where a vortex forms due to the interaction of the flow from the graft with the relatively slow flow in the occluded proximal artery, whose size changes with the flow phase (see Figure 8b). The presence of a slow recirculation flow (i.e. a vortex) increases the near-wall residence time and results in platelet activation [109] and fibrin thrombus formation [110,111], which leads to IH development [14,17,83,88,97,98].

Along with this vortex, there is a stagnation point on the artery bed, where the graft flow impinges the floor (Figure 8b), whose location oscillates (with the size of the vortex) during the cardiac cycle. This moving stagnation point provides a low-magnitude‒high-oscillatory WSS condition on the artery bed which is prone to enhancement of atherogenesis [100] and IH formation [102,112]. In addition, the flow impact on the artery floor is known to be injurious to the endothelium and is believed to be a contributing factor to the graft failure, as there is evidence of change in the flow character once it impacts against the junction floor [113].

**Figure 9:**
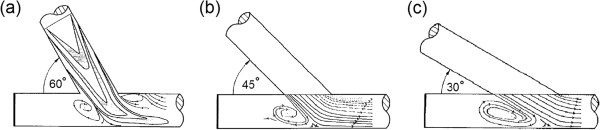
**Effect of anastomotic angle on the flow regime.** Flow streamlines in the symmetry plane of distal ETS anastomoses with different anastomotic angles **(a)** 60°, **(b)** 45°, and **(c)** 30°. Flow separation at the toe and size of the reversed-flow region downstream of the anastomosis increases with anastomotic angle (taken from [27] with permission).

In a conventional ETS configuration, there are high flow shear rates at the toe of the anastomosis (causing high WSS at the toe), usually with a flow separation region just distal to the toe at the inner wall of the coronary artery (causing flow recirculation and low WSS at this area), as shown in Figure 9a. This results in a high spatial gradient of WSS at the toe of the anastomosis, which induces morphological and functional changes in the endothelium that contribute to elevated wall permeability and consequent atherosclerotic lesions [105,107,108] and IH development [84]. Moreover, in flow separation and flow recirculation zones, due to low WSS and enhanced particle residence time, excess cholesterol is deposited on the surface of the lumen, initiating atheroma growth [92] and IH [23,109].

Although an ETS anastomosis is essentially a bifurcation, being manmade, surgically created anastomoses can be modified (in contrast to arterial bifurcations) to yield a flow environment that improves graft longevity [45]. As reviewed above, investigations of blood flow and HPs and their comparison with focal locations of IT and IH formation in CABGs have resulted in correlation of some HPs with initiation and progression of IH. Consequently, HPs can, in turn, be utilized as indicators to show susceptible sites of IT and favorable conditions for thrombi and IH formation. Accordingly, using these indicators (i.e., by modification of HPs), extensive efforts have been put to obtain an optimal graft design, which is an end point for the study of correlations between hemodynamics and graft failure.

### Attempts to design an optimal anastomotic configuration

The first efforts towards attaining an optimal anastomosis have been made by ***changing the anastomotic angle***. It has been shown that tissue remodeling at ETS arterial anastomoses is highly sensitive to graft angle [24], and graft patency rates vary according to anastomotic angle. The anastomotic angle affects the flow regime and shear stress [21,25]. A smaller anastomotic angle reduces (i) the peaks and gradients of WSS [21], (ii) the flow separation and disturbances at the toe [22,23,26,27], (iii) secondary flow components [23], and (iv) size of recirculation area (i.e., reversed flow) downstream of the anastomosis [25-27], as shown in Figure 9. Hence, a smaller distal ETS anastomotic angle (≤ 30°) seems to bring about a less disturbed and more uniform, smooth flow from the graft into the coronary artery.

***The effect of graft caliber*** (i.e., graft-to-host diameter ratio) on the hemodynamics of CABGs has also been examined by investigators. It is observed that larger graft-to-host diameter ratios (5:3) have better hemodynamic performance than smaller ones (1:1) [32], as they can bring about relatively large positive longitudinal velocity, uniform and large WSS [33], and small WSSG [21,33]. Likewise, results of a computational study, using mesh-less CFD and genetic algorithms optimization, indicate that the graft caliber should always be maximized, in order to minimize the spatial and temporal gradients of WSS [34].

Besides, smaller grafts typically present an increased risk of early graft failure due to thrombosis [114]. Several clinical studies have demonstrated that small caliber (<3.5 mm) of vein grafts is the only independent risk factor for vein graft stenosis [115,116]. Idu et al. [115] suggested that a small caliber is a greater risk factor for graft failure than the use of arm or composite vein grafts, and that these alternative veins should be preferred if the saphenous vein graft is less than 3.5 mm in diameter.

It has been observed that a small-diameter (<3 mm) saphenous vein graft is associated with a 2.1-fold increased risk of early failure [117], and such conduits have a higher rate of occlusion in the perioperative (0‒30 day) interval [118]. Moreover, observations from a large multi-centre trial suggest that small size of vein graft is the dominant technical determinant of early graft failure [117].

In addition, a smaller graft diameter increases the graft resistance against the flow, which can elevate the flow portion through the native (partially stenosed) coronary and escalate the competitive flow problem [119] that eventually results in graft thrombosis and failure.

***The effect of competitive flow*** (i.e., flow through a bypassed native coronary artery with low degree of stenosis) on the graft patency has been extensively investigated, but still is somewhat controversial. Many studies have demonstrated that the patency of bypass grafts on functionally significant lesions is considerably higher than the patency of bypass grafts on non-significant lesions [120-127]. They have confirmed the existence of a critical value for stenosis severity, below which the graft failure is expected, and above that, the recipient artery will be progressively occluded [119,128].

Although competitive flow (from patent native coronary vessels) is implicated in the failure of internal mammary artery (IMA) grafts, it is not thought to affect the patency of saphenous vein grafts (SVGs) [120,124,129]. This is because non-muscular SVGs cannot adjust their lumens in response to metabolic requirements as much as arterial grafts. Thus, the response of vein grafts to low flow is limited [123].

On the other hand, some studies have demonstrated that despite of significant correlation between (low) degree of proximal stenosis of the recipient coronary artery (i.e., presence of competitive flow) and occurrence of a string sign (where the graft conduit is patent but with only a thread of antegrade flow, due to narrowing of the graft), chronic native competitive flow does not significantly affect midterm graft status [130] and that the flow rates of the IMA grafts are comparable with and without stenosis or string phenomenon [131]. Also, limited studies have reported that competitive flow from a moderately stenotic coronary artery has not predisposed patients toward the string sign of the IMA graft in the presence of substantial diastolic IMA flow [132].

***The effects of stenosis severity and distance of grafting*** on the hemodynamics of distal anastomosis have also been investigated. Computational simulations have shown that in the case of bypass grafting of partially stenosed coronary artery, the flow through partially occluded host artery interacts with the bypass graft flow at the anastomotic junction and that this combined flow can cause adverse hemodynamic effects, particularly when the distance of grafting is short [39,133-135]. The jet flow from a partially stenosed artery can increase the peak value of the axial velocity, if the stenosis is close to the anastomosis [134]. Also, interaction between the flows from the graft and the partially occluded artery results in steep variations of WSS near the heel and toe of the anastomosis, which can facilitate intimal proliferation and thrombogenesis around the suture-line when combined with flow recirculation in these regions [134]. Thus, it has been recommend that anastomosis be sutured with a sufficient distance of grafting, to enable the velocity profile to fully reattach before the heel so as to minimize the risks of intimal hyperplasia at the anastomosis [133].

**Figure 10:**
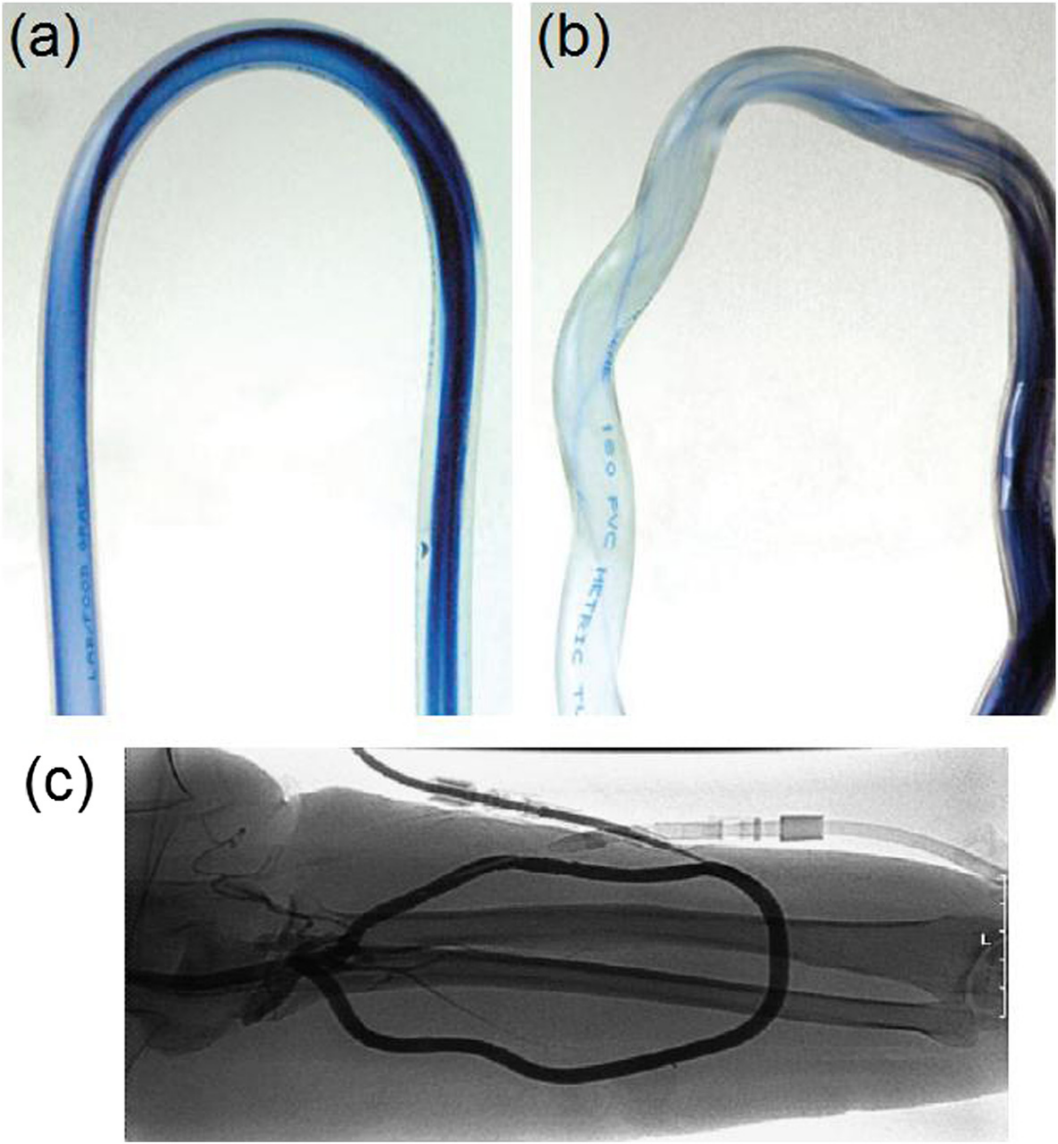
**Helical graft.** Flow mixing visualization by bolus injection into water flow (Re = 550) in U-tubes with **(a)** a conventional tube, and **(b)** a helical tube. Significantly greater mixing can be observed in the helical tube, which can enhance fluid-wall mass transport and render the spatial distribution of WSS relatively uniform in curved conduits. **(c)** angiogram of an arteriovenous access PTFE helical graft. Angiographic examinations have suggested that there exists reduction of helical geometry at or after implantation, which might be attributable to graft elongation under arterial pressure (taken from [136,137] with permission).

***The influence of out-of-plane graft curvature*** has been studied by several investigators [36]. These investigations have revealed reductions in magnitudes of the peak time-averaged WSS [37] and mean oscillatory shear [35] in the non-planar models as compared to the planar configurations, which imply a corresponding reduction in the spatial extent of wall regions exposed to physiologically unfavorable flow conditions [35]. Accordingly, in order to induce non-planar flow effects, the use of grafts with intrinsic helical axis was suggested [136]. In vitro flow visualizations have shown significantly increased cross-plane mixing for the helical grafts (Figure 10a and b), and preliminary in vivo studies in arteriovenous bypass grafts have indicated that helical grafts offer the potential for improved patency (Figure 10c) [136,137]. This can be attributed to the swirling flow effects induced by helical grafts which increase the magnitudes of velocity and WSS by adding a secondary (circumferential) velocity component to the axial velocity. This can enhance fluid-wall mass transport and render the spatial distribution of WSS relatively uniform in curved conduits, and potentially at anastomoses [136].

Nevertheless, the graft non-planarity is often constrained by surgical considerations beyond hemodynamics (e.g., the stenosed artery, location of stenosis, etc.) [86].

**Figure 11:**
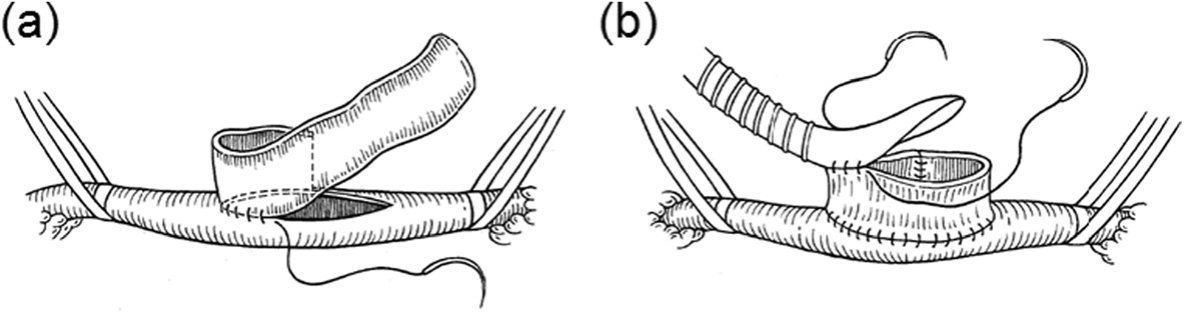
**Miller cuff construction. (a)** Vein cuff is sewn longitudinally around arteriotomy **(b)** graft is then sutured end-to-cuff. Using a cuff has adverse effects on hemodynamic factors around the anastomosis (e.g., large variations in shear stress on the artery floor, low-momentum recirculation within the cuff, and prominent separation at the cuff toe), and any improved patency rates achieved with cuffed anastomoses have been attributed to increased anastomotic volume and the consequent ability to accommodate IH, before it causes significant stenosis, rather than any decrease in IH (taken from [139] with permission).

Further efforts have been put to obtain a more favorable anastomosis by ***design of cuffed and patched anastomotic configurations***. Miller et al. [138] introduced a vein cuff design that produced good patency in femoral-distal grafts (Figure 11). However, the utility of this technique is somewhat controversial. While some studies found that grafts implanted with vein cuffs resulted in decreased developments of IH [140,141] and had better patency than those with a non-cuffed anastomosis [142], other investigations showed that (i) the use of a Miller cuff caused no difference in IH thickness [143], (ii) the improved patency was only in below-knee grafts (and not in above-knee popliteal bypasses) [138,139,144], and (iii) the use of a cuff has adverse effects on hemodynamic factors around the anastomosis, such as large variations in shear stress on the artery floor, low-momentum recirculation within the cuff, and prominent separation at the cuff toe [145-147]. It is hence suggested that the improved patency rates achieved with cuffed anastomoses are due not to a decrease in IH but to an increased anastomotic volume and the consequent ability to accommodate IH, before it causes significant stenosis [143,148].

**Figure 12:**
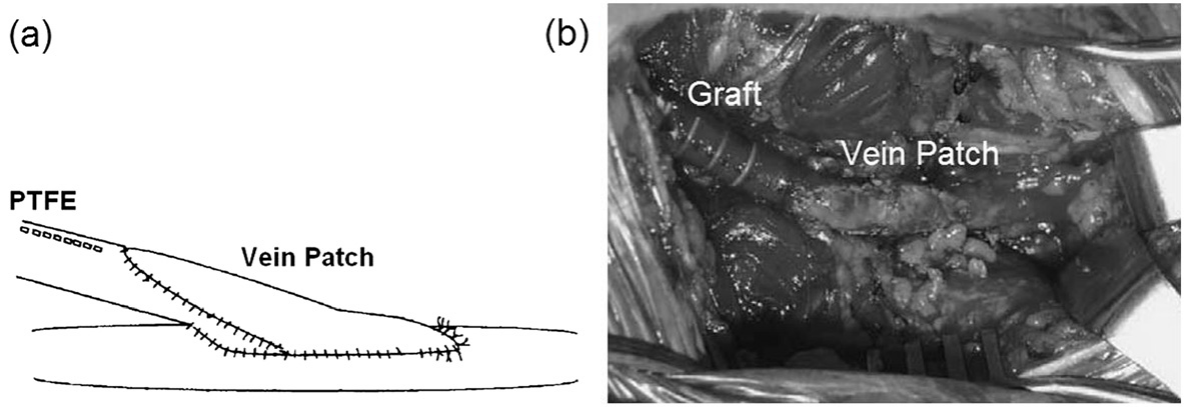
**Taylor vein patch. (a)** Schematic drawing of Taylor-patched anastomosis. **(b)** Intraoperative photograph of distal Taylor vein patch (6 mm PTFE graft bypass to below knee popliteal artery). Patched grafts have not shown significant improvement in primary patency rates as compared to non-patched grafts (taken from [150,151] with permission).

The Taylor vein patch technique [149] (Figure 12) has been found to decrease IH [150], diminish flow disturbances and undesirable flow separation at the toe of the anastomosis [28], and slightly reduce the WSSG in the anastomotic region [29]. However, its improvement in hemodynamic factors is minor. Besides, patched and non-patched grafts have shown similar primary patency rates [150].

**Figure 13:**
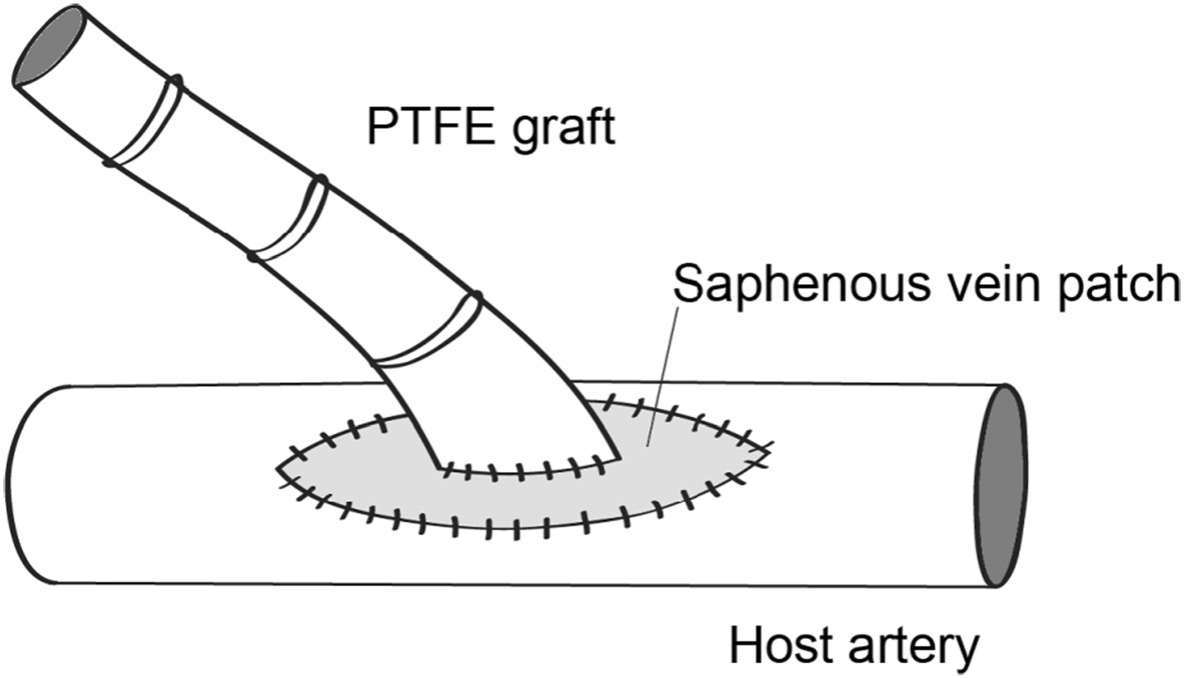
**Linton patch.** Schematic drawing of a Linton-patched anastomosis. The flow patterns of patched grafts are similar to those of conventional ETS anastomosis. The clinical patency of this technique has been reported to be 65-74% at 12-48 months post-operative.

Linton patch was introduced as a technique in which the conduit is patched with a venous segment of about 40-50 mm long [152], as shown in Figure 13, and it was frequently used in femoral artery to facilitate construction of the proximal anastomosis of femoropopliteal bypasses. Linton patch technique could considerably increase the compliance at the junction. However, its flow patterns have been shown to be similar to those of conventional ETS anastomosis. The clinical patency of this technique has been reported to be 65-74% at 12-48 months post-operative [153].

**Figure 14:**
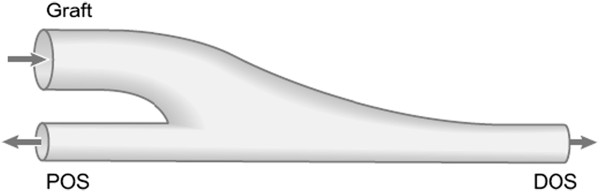
**Lei’s improved anastomotic geometry.** Improved anastomotic geometry with S-shaped gradual transition in wall curvature and cross-sectional area at the toe region results in significant reduction of WSSG at the toe and on the floor as compared with standard ETS and Taylor patched configurations (adopted from [29] with permission).

Lei et al. [29], utilizing an iterative optimization procedure coupled with CFD simulations, further improved the geometric design of the Taylor patch to obtain smaller WSSGs. This improved design, whose anastomotic surface area was smaller than that of the Taylor patch, yielded a significant reduction in local time-averaged WSSG (ranging from two- to six-fold decrease, compared with standard and Taylor hooded configurations for a variety of flow splits between POS and DOS) both at the toe and on the floor. This reduction was due to the gradual S-shaped transition in wall curvature and cross-sectional area at the toe region, as shown in Figure 14.

**Figure 15:**
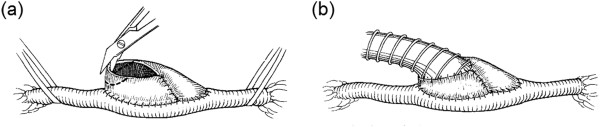
**Tyrell vein collar.** Diagram of a Tyrell vein collar **(a)** without and **(b)** with a PTFE graft. Trials of Tyrrell collar venous anastomosis in AVGs have not shown any improvement in graft patency (taken from [154] with permission).

The Tyrrell collar has been developed in attempts to incorporate the advantages of the Miller and Taylor anastomotic designs, by avoiding direct suturing of the graft and artery (which can cause high compliance mismatch in case a synthetic graft is used), and providing a more streamlined shape at the toe [154] (Figure 15). However, trials of Tyrrell collar venous anastomosis in arteriovenous grafts (AVGs) not only showed no improvements in graft patency [155], but also indicated that the use of the collar at the venous anastomosis of forearm loop AVGs resulted in early graft failure [156].

**Figure 16:**
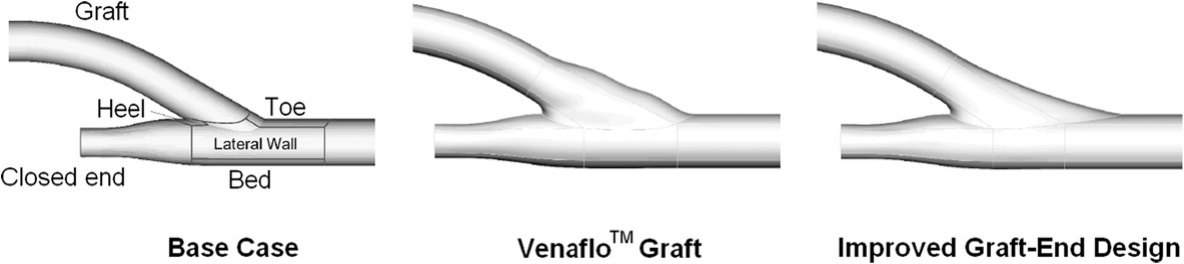
**Numerical studies of three ETS anastomotic configurations.** Geometric models of a conventional ETS anastomosis (base case), Venaflo™ graft, and modified graft-end design. Venaflo™ graft provides larger junction area and less disturbed flow patterns than the conventional ETS anastomosis, and the modified graft-end design further reduces the WSSG by elimination of the graft bulges. Results of clinical trials of the Venaflo™ graft are controversial; some studies showed promising graft patency rates in the Venaflo™ grafts (58% versus 21% in the conventional standard grafts at 24 months), while other investigations demonstrated inferior 1-year patency rates of the Venaflo™ grafts (43% versus 47% for non-cuffed ePTFE grafts) (adopted from [31] with permission).

Longest and Kleinstreuer [31] numerically simulated the haemodynamics for a conventional ETS anastomosis (as the base case), the Venaflo™ graft, and an improved cuffed graft-end configuration for AVGs (Figure 16). The Venaflo™ graft demonstrated considerable improvements over the base case by enlarging the junction area and reducing the severity of disturbed flow patterns in predictive computer simulations. Considering the critical toe region, further improvements were achieved in the modified graft-end design by smoother wall curvatures and elimination of the graft bulges, which further reduced the maximum normalized WSSG to 6.4 from 18.1 for the Venaflo™ graft. However, results of clinical trials of the Venaflo™ graft have been controversial. Some studies have shown promising graft patency rates in the Venaflo™ grafts (58% versus 21% in the conventional standard grafts at 24 months) [157], while other investigations have demonstrated the 1-year patency rates of the Venaflo™ grafts to be inferior to those of non-cuffed ePTFE grafts (43% versus 47%) [158].

**Figure 17:**
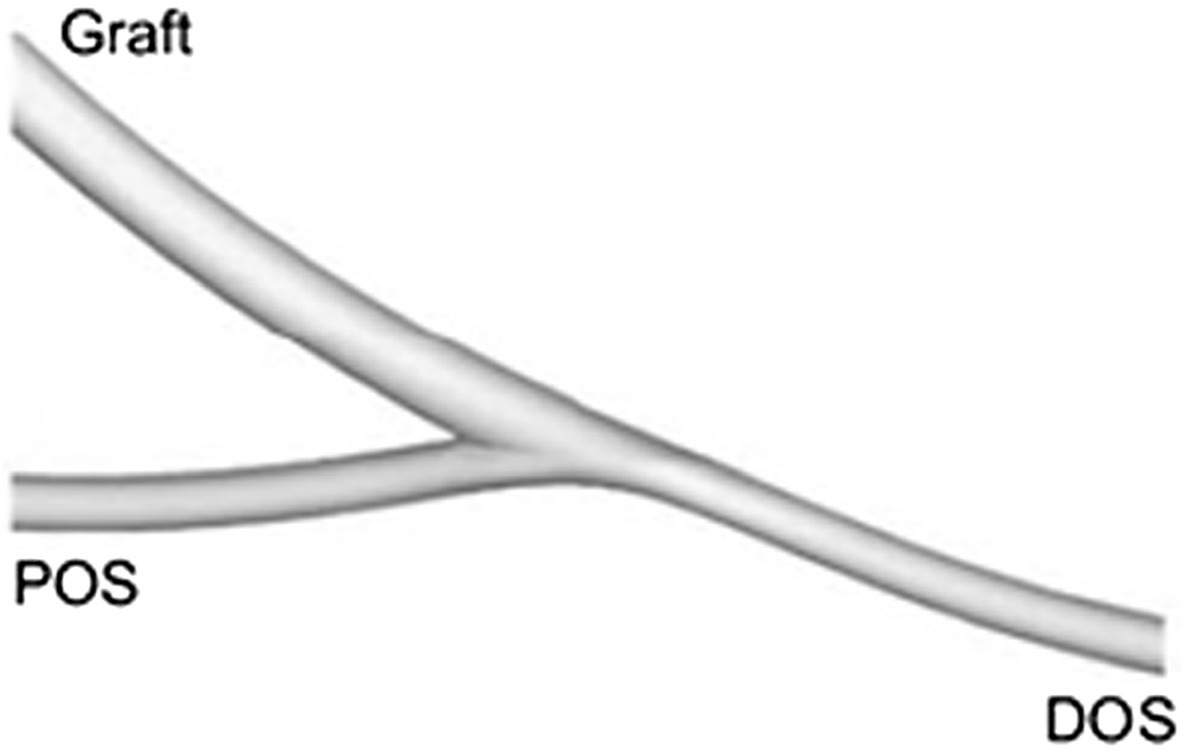
**Streamlined anastomotic configuration.** Streamlined arterial bypass graft configuration: Although this geometric design can reduce the peak WSSG at the toe of the anastomosis, particle-wall interaction remains significant, which can result in platelet activation and may lead to IH (adopted from [159] with permission).

A streamlined anastomotic configuration in which the distal outlet segment (DOS) is aligned with the graft has been developed by Longest et al. [159]. This configuration, shown in Figure 17, resulted in an advantageous reduction of the peak normalized WSSG values in the vicinity of the toe (to 1.7 from 11.8 in a conventional ETS model). However, particle-wall interactions remained significant throughout the anastomosis, which can result in platelet activation and may lead to IH.

**Figure 18:**
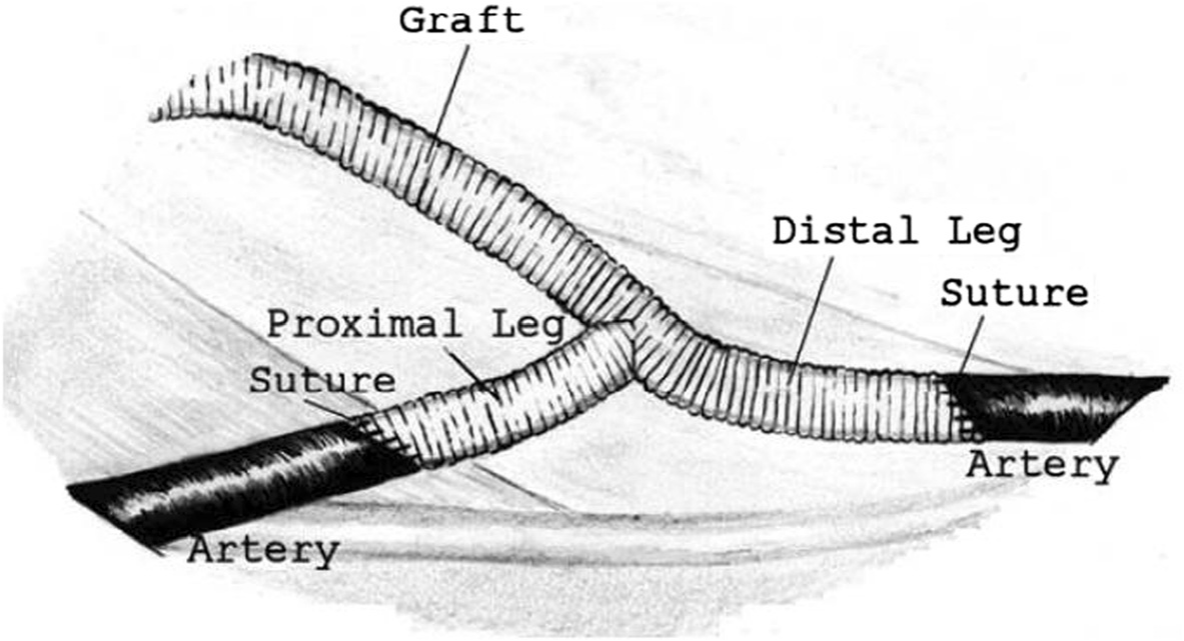
**Prosthetic bifurcating graft-end configuration.** Schematic drawing of the prosthetic graft-end configuration designed to reduce flow stagnation and flow separation zones. This prosthetic graft can be connected in an ETE fashion with the POS and DOS (adopted from [160] with permission).

O’Brien et al. [160] have designed a configuration to replace the anastomosis with a synthetic bifurcation connected in an ETE fashion with the proximal outlet segment (POS) and DOS (Figure 18). Their numerical simulations indicated that the smoothly curving bifurcation improves the WSS environment by reducing flow separation and stagnation. Although this prosthetic graft configuration has primarily been designed for femoral-popliteal bypasses, the concept may be relevant in other aspects of cardiovascular surgery. This prosthetic graft can be manufactured from clinically proven synthetic materials, does not require any additional training in its use, and combines attributes of ETS anastomoses with those of ETE anastomoses.

**Figure 19:**
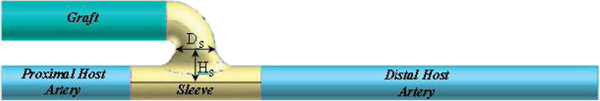
**Cuff-like sleeve.** Schematic view of a distal ETS anastomosis with an incorporated sleeve. Sleeve models with higher necks were deemed preferred in terms of hemodynamics at the distal anastomosis (taken from [161] with permission).

Chua et al. [161] designed a cuff-like sleeve for implantation at the distal anastomosis of CABGs as a connector between the graft and the host artery (Figure 19). Their computational simulation results suggested that the sleeve models with higher necks were preferred in terms of hemodynamics at the distal anastomosis.

**Figure 20:**
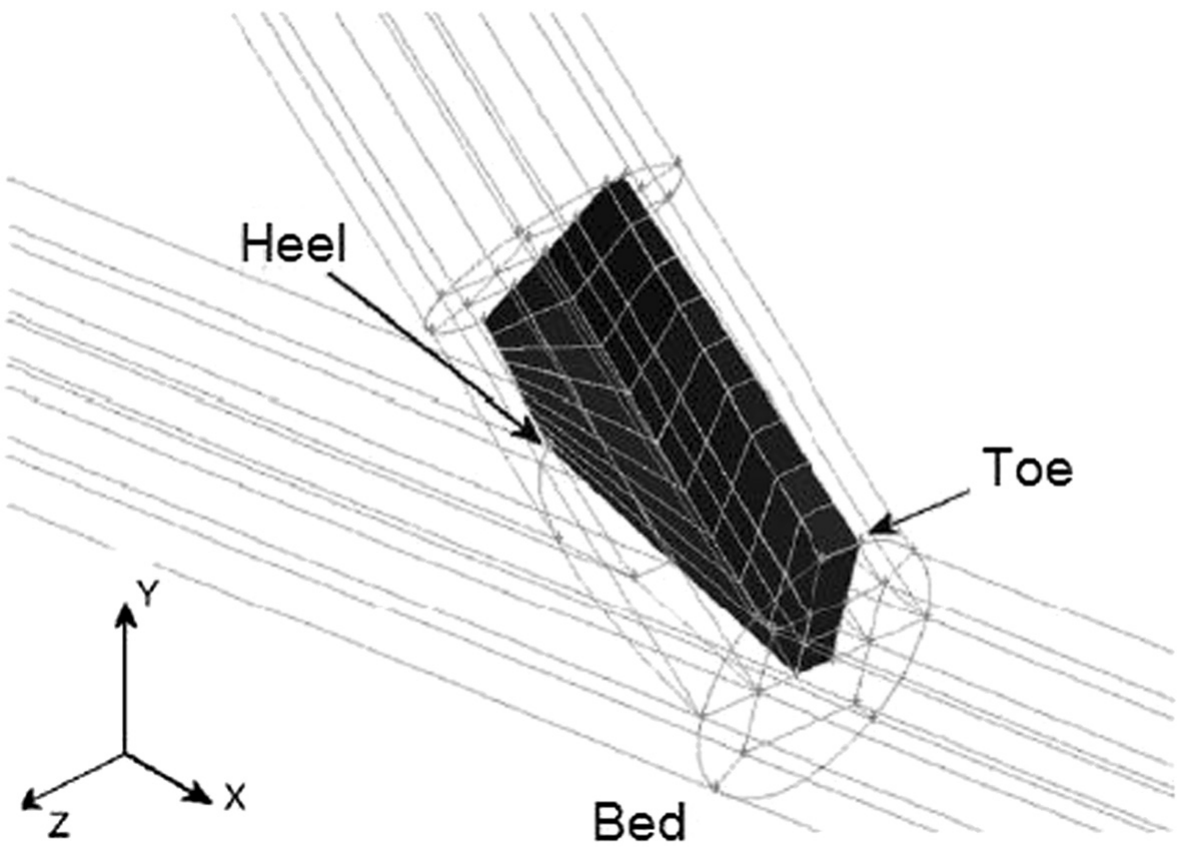
**Flow-splitter.** Schematic wire-frame view of a graft-artery junction and implanted flow-splitter. This flow-splitter can divert the flow from the arterial bed to avoid flow impingement on the bed, mitigate the flow separation at the toe, and reduce the size of flow recirculation areas. However, it causes flow impingements on the arterial side-walls and increases the WSS on both sides of the artery bed centerline which can result in high values of time-averaged WSSG (taken from [162] with permission).

In an attempt to alter the disturbed hemodynamic on the artery bed in the ETS anastomosis, O’Brien et al. [162] designed a flow-splitter to be placed into the junction of distal ETS anastomosis, as shown in Figure 20. This flow-splitter splits the flow profile entering the anastomosis into two channels and diverts the flow from artery bed toward the arterial side-walls. Although this flow-splitter could reduce the peaks of WSS and WSSG on the bed (by 36% and 49%, respectively) at particular phases (during deceleration) and also mitigate the flow separation at the toe, it caused large increases in WSS on both sides of the artery bed centerline which can result in high values of time-averaged WSSG over the cardiac cycle near the centerline on the arterial bed. Besides, implantation of this flow-splitter may be practical only if integrated in a synthetic graft suite, and not along with autologous grafts.

**Figure 21:**
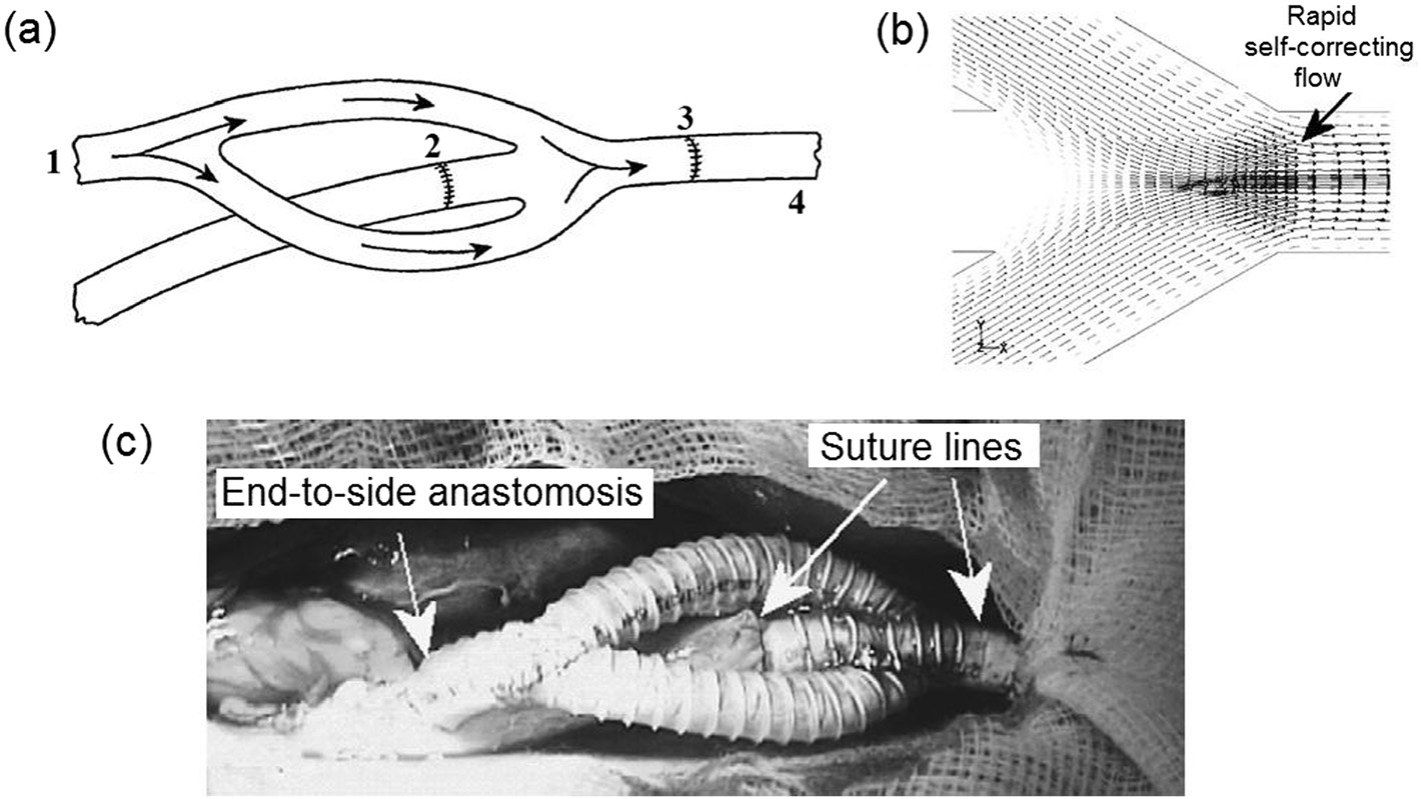
**Prosthetic bifurcating vascular grafting device. (a)**: (1) Flow from the proximal anastomosis (2,3) suture-lines of the end-to-end anastomoses of the distal section (4) the host artery. **(b)** The bifurcated flows impinge upon each other at the central lumen of the distal anastomosis and avoid arterial bed impingement to reduce the possibility of IH formation. Also, flow separation at the toe is eliminated and the opposing self-correcting flows rapidly return to normal hemodynamic behavior. **(c)** Intraoperative photograph of the graft implanted into a porcine aorta (adopted from [163,164] with permission).

Walsh et al. [163] have designed a novel vascular grafting device with a bifurcating configuration (Figure 21a), in order to eliminate the flow impingement on the interior wall of the artery at the distal anastomosis. In this vascular device, the flow from the proximal anastomosis is bifurcated into two branches and these branch flows impinge upon each other at a central region of the lumen at the distal anastomosis. By avoiding arterial bed impingement, the possibility of disease formation is reduced. Besides, the opposing branch flows rapidly regain the normal hemodynamic behavior in the distal artery (Figure 21b). Another positive feature of this design is the mitigation of flow separation at the toe. This prosthetic vascular graft can be incorporated into the host artery by means of two ETE anastomoses (as shown by suture-lines 2 and 3 in Figure 21a) at the distal section and a side-to-end anastomosis at the proximal section (not shown here). Surgical feasibility of this design for treatment of peripheral arterial disease has been verified in vivo, by implantation of a PTFE graft into the aorta of a pig model (Figure 21c) [164]. However, a major limitation of this graft is its geometrical complexity.

#### Coupled sequential anastomoses design

**Figure 22:**
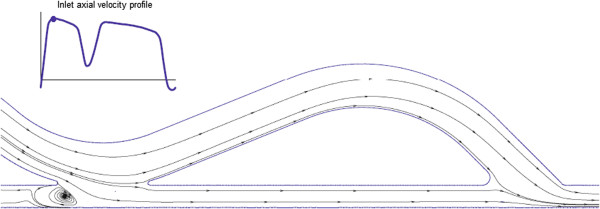
**Coupled STS-ETS sequential anastomoses bypass graft design.** Flow streamlines through the coupled STS-ETS sequential anastomoses bypass graft design. Part of the graft flow, which is diverted into the coronary artery at the STS anastomosis, lifts up the flow coming from the graft at the ETS anastomosis and directs it smoothly into the coronary artery; this prevents arterial bed impingement and eliminates the stagnation point and flow recirculation at the ETS anastomosis. This design provides a spare route for the blood flow to the coronary artery to avoid re-operation in case of re-stenosis in either of the anastomoses (adopted from [167] with permission).

Based on the advantageous flow characteristics observed within the side-to-side (STS) anastomosis of typical sequential bypass grafts (i.e., a smoother flow with smaller spatial gradients of WSS than those in an ETS anastomosis [165]) and higher patency rates in STS anastomoses than in ETS anastomoses [166], Kabinejadian et al. [167,168] developed a novel coupled STS-ETS sequential anastomoses bypass graft design, as shown in Figure 22. In this design, part of the graft flow is diverted into the coronary artery at the STS anastomosis, and when this flow in the coronary artery reaches the ETS anastomosis, it lifts up the flow coming from the graft and directs the graft flow smoothly into the coronary artery, which prevents impingement of blood flow on the arterial bed and eliminates the stagnation point and flow recirculation at the ETS anastomosis.

Computational simulations of blood flow through this novel design have shown improvements of HPs, especially at the heel and on the arterial bed of the ETS anastomosis. These improvements in distribution of HPs include an increase in the time-averaged WSS on the artery bed of the ETS anastomosis of the SQA (as compared to the conventional ETS anastomosis), reduction of the time-averaged WSSG at the heel and bed of the ETS component as well as at the toe and suture line of the STS component of the novel SQA (as compared to the conventional ETS and typical parallel STS anastomoses, respectively), and reduction of the OSI at the ETS anastomosis of the SQA at the heel region and on the artery wall and bed opposite to the heel (in comparison with the conventional ETS anastomosis). Besides, this design provides a spare route for the blood flow to the coronary artery in order to avoid re-operation in case of re-stenosis in either of the anastomoses. This design can be employed using autologous grafts without the need for any additional training.

## Conclusions and future directions

The search for an ideal distal anastomotic configuration for coronary bypass grafting has led to numerous designs. Optimal anastomosis design must take into account practical issues such as surgical construction. An anastomotic design should be feasible to be implemented by surgeons in a reasonable time.

As reviewed, some of the designed anastomotic configurations which are feasible to be constructed during operation using autologous materials (including Miller cuff, Taylor patch, and Tyrrell collar) have not shown a remarkable enhancement in patency of bypass grafts. On the other hand, some configurations which have shown considerable improvement in HPs distribution can only be made by synthetic materials due to their complex geometry (such as Venaflo™ graft, bifurcating graft-end design, cuff-like sleeve, and bifurcating vascular graft); this has consequences of blood-exposed non-intimal surface and high compliance mismatch at the synthetic material-blood vessel interface.

Moreover, the anastomotic designs which have improved HPs distribution and are feasible to be surgically constructed by autologous grafts (such as the coupled sequential anastomoses design), have not gone through animal trials or clinical investigations yet to demonstrate their in vivo performance and patency rates. Hence, the dilemma of designing an optimal anastomosis, which can bring about considerable improvements in the flow regime, HPs distribution, and graft patency, still remains unsolved.

In conclusion, there are a few aspects to be considered in the design of an optimal CABG:

(i) *Compatibility of the graft with the arterial pressure and the supplied blood flow rate*, to ensure a physiologic range of intramural stresses and hemodynamic forces in the graft itself. Arterial grafts, such as left internal mammary artery (LIMA), have demonstrated considerably higher patency rates than the most commonly used saphenous vein grafts [169]. However, due to lack of arterial conduits, veins are currently used most commonly as grafts. With technological advances, the time required for production of matured implantable tissue-engineered grafts, which could fulfill the ideal characteristics present in the arteries, will be shortened [170] and they can replace the vein grafts in CABG.

(ii) *Arterial compliance of the graft*, to avoid compliance mismatch with the host artery at the anastomotic junction, to prevent escalation of intramural stresses in the artery and the graft, which can result in IH formation, especially on the suture-line. As discussed in this review, compliance mismatch between the graft and the host artery results in an increase of intramural stresses, which in turn promotes IH. Use of arterial conduits can (to some extent) address this issue too. However, as mentioned above, tissue-engineered grafts might be the future solution to this problem.

(iii) *Hemodynamic performance driven design of anastomotic configuration of the distal anastomosis*, to regulate the hemodynamic parameters and wall shear stress indices, in order to avoid triggering of the pathogenic factors of IH and thrombosis (e.g., platelet activation, long near-wall residence time, etc.). As reviewed, it is well established that HPs play an important role in the initiation and progression of atherosclerosis and IH. A hemodynamically optimized anastomotic configuration can provide moderate shear stress parameters and smooth blood flow without flow disturbances, to avoid triggering the associated atherogenic phenomena.

(iv) *Minimal vascular injury*, to minimize proliferation of SMCs as a wound healing response. Technological advances may further develop the suggested alternatives to sutures (e.g., biological glues, laser generated solders, etc.) to a practicable level for routine clinical use. Not only can such products minimize vascular injury, but also they can eliminate the para-anastomotic hyper-compliant zone and the associated elevating intramural stresses which are caused by the stiff sutures.

(v) *Patient-specific designs*, to tailor the design considerations to each particular patient’s cardiovascular characteristics. Development of clinical imaging (e.g., magnetic resonance and computed tomography) enables a detailed patient-specific description of the actual hemodynamics and structural behavior of living tissues. Coupling of these data with engineering analyses is becoming a standard evaluation that is expected to become part of the clinical practice in diagnosis and surgical planning in advanced medical centers [171]. This would optimize the design considerations and choice of graft for each particular patient, depending on the number, location, and severity of stenosis, etc.

## Abbreviations

AVG: Arteriovenous graft; CABG: Coronary arterial bypass grafting; CAD: Coronary artery disease; CFD: Computational fluid dynamic; DAIH: Distal anastomotic intimal hyperplasia; DOS: Distal outlet segment; EC: Endothelial cell; ETE: End-to-end; ETS: End-to-side; HP: Hemodynamic parameter; IH: Intimal Hyperplasia; IMA: Internal mammary artery; IT: Intimal thickening; LAD: Left anterior descending coronary artery; LDL: Low-density lipoprotein; LMCA: Left main coronary artery; OSI: Oscillatory shear index; PCI: Percutaneous coronary intervention; POS: Proximal outlet segment; RCA: Right coronary artery; SMC: Smooth muscle cell; STS: Side-to-side; SVG: Saphenous vein graft; WSS: Wall shear stress; WSSG: Wall shear stress gradient.

## Competing interests

The authors declare no competing interest with regards to this invited review.

## Authors’ contribution

FK: collection, organizing, and review of the literature; preparing the manuscript. DNG: manuscript review, modification, editing, and revision. Both authors read and approved the final manuscript.

## Authors’ information

DNG: He is currently involved with **Southern Ozarks Alliance for Rural Development in Willow Springs MO**, setting up the Ozark Rural University MO (USA), and advising universities in their Programs development. He is a pioneer in the fields of biomedical engineering, healthcare engineering, and management. He has published over 450 works in the fields of engineering science, biomedical engineering, medical science, and social sciences, as well as 29 books on biomedical engineering, engineering physiology, cardiovascular physics, orthopedic mechanics, medical and life physics, and spinal injury. The inventor of life-saving implant devices has also been the former Chief Academic Officer (Provost) & Registrar of the Parkway College of Health Sciences (Singapore) and Professor in the School of Chemical & Biomedical Engineering of Nanyang Technological University (Singapore).

FK: He is currently a postdoctoral research fellow at **National University of Singapore (NUS)**. He has about 10 years of research experience in Biomedical Engineering, including analytical, experimental, and computational investigations on biofluid and cardiovascular mechanics and design and development of relevant medical devices.
